# Encapsulation Engineering of Sulfur into Magnesium Oxide for High Energy Density Li–S Batteries

**DOI:** 10.3390/molecules29215116

**Published:** 2024-10-30

**Authors:** Sunny Choudhary, Nischal Oli, Shweta Shweta, Satyam Kumar, Mohan K. Bhattarai, Carlos Alberto Malca-Reyes, Rajesh K. Katiyar, Balram Tripathi, Liz M. Díaz-Vázquez, Gerardo Morell, Ram S. Katiyar

**Affiliations:** 1 Department of Physics, University of Puerto Rico at Río Piedras, San Juan, PR 00931, USA; 2 Department of Chemistry, University of Puerto Rico at Río Piedras, San Juan, PR 00931, USA; 3 Department of Physics, S.S. Jain Subodh P.G. (Autonomous) College, Jaipur Rajasthan 302004, India

**Keywords:** vibrational modes in Li–S, sulfur–MgO composite, energy storage applications, cyclic stability, morphology sulfur cathode, polysulfides

## Abstract

This study addresses the persistent challenge of polysulfide dissolution in lithium–sulfur (Li–S) batteries by introducing magnesium oxide (MgO) nanoparticles as a novel additive. MgO was integrated with sulfur using a scalable process involving solid-state melt diffusion treatment followed by planetary ball milling. XRD measurements confirmed that sulfur (S_8_) retains its orthorhombic crystalline structure (space group $F_{ddd}$ ) following the MgO incorporation, with minimal peak shifts indicating slight lattice distortion, while the increased peak intensity suggests enhanced crystallinity due to MgO acting as a nucleation site. Additionally, Raman spectroscopy demonstrated sulfur’s characteristic vibrational modes consistent with group theory (point group $D_{2}h$ ) and highlighted multiwalled carbon nanotube (MWCNT′s) D, G, and 2D bands, with a low I_D_/I_G_ ratio (0.47), which indicated low defects and high crystallinity in the prepared cathode. The S–MgO composite cathode exhibited superior electrochemical behavior, with an initial discharge capacity (950 mA h g^−1^ at 0.1 C), significantly improved compared to pristine sulfur’s. The presence of MgO effectively mitigated the polysulfide shuttle effect by trapping polysulfides, leading to enhanced stability over 400 cycles and the consistent coulombic efficiency of over 99.5%. After 400 cycles, EDS and SEM analyses confirmed the structural integrity of the electrode, with only minor fractures and slight sulfur content loss. Electrochemical impedance spectroscopy further confirmed the enhanced performance.

## 1. Introduction

**Figure 1:**
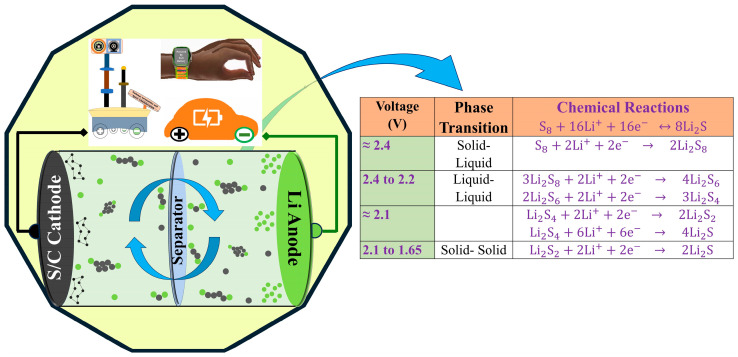
Schematic of Li–S battery and Electrochemical classification.

Lithium–sulfur (Li–S) batteries are expected to be the basis for next-generation high-energy rechargeable batteries due to their high theoretical specific capacity (1673 mA h g^−1^), high theoretical specific energy (2567 Wh kg^−1^), and low cost. Although the sulfur cathode has recently been operated beyond the topotactic discharge voltage for Li–S to obtain high capacities of around 900 mA h g^−1^ and more, their applications are hindered by several technical challenges [1,2,3], including rapid capacity fading, low coulombic efficiency, and irreversible active mass loss. An effective way to overcome these challenges is to bind the Li_2_S_x_ species to host materials in order to suppress the polysulfide shuttle, thus enhancing the cycling stability of the Li–S batteries. An ideal anchoring material should (1) exhibit robust but not too strong of a chemical interaction with the Li_2_S_x_ species (0.8–2.0 eV including Van der Waals interactions) to effectively trap them; (2) keep the species intact to prevent their dissolution into the electrolyte; (3) have a large and sufficient active region to accommodate sulfur volumetric expansion; and (4) possess a small characteristic dimension of the sulfur electrode to avoid pulverization. Therefore, it is imperative to control the volume fluctuations of sulfur upon cycling, suppress the dissolution of the intermediate polysulfides, and improve the ionic/electronic conductivities of sulfur and relevant metal sulfides [4,5,6,7,8]. Figure 1 shows the schematic and electrochemical reaction mechanism of the Li–S batteries.

The theoretical density of sulfur is about 2.07 g cm^−3^, notably lower than that of the typical cathode materials in conventional Li–ion batteries, LiCoO_2_ (5.1 g cm^−3^), LiNi_x_Co_x_Mn_x_O_2_ NMC (4.8–5.3 g cm^−3^), and LiMn_2_O_4_ LMO (4.3 g cm^−3^). To enhance the volumetric capacity of sulfur cathodes, it is essential to utilize host materials that possess higher density and improved functionality. Transition metal oxides, exhibiting densities between 3.5 and 5.1 g cm^–3^, provide a significant benefit in enhancing the overall volumetric capacity. Magnesium oxide (MgO), possessing a theoretical density of 3.58 g cm^−3^, serves as an appropriate additive for sulfur–based cathodes, facilitating an optimal balance between high density and functionality. The substantial specific surface area (150–300 m^2^ g^−1^), resulting from its nanostructured form, enhances the cycling stability. The interaction of MgO with lithium polysulfides (LiPSs) improves structural integrity, facilitates the conversion of LiPS, and reduces the shuttle effect, resulting in enhanced electrochemical performance [9,10].

Moreover, the incorporation of functional groups [11,12,13,14] such as oxygen, nitrogen, boron, and sulfur into carbon structures has demonstrated an improvement in polysulfide adsorption via both chemical bonding and physical confinement. This dual interaction diminishes the shuttle effect considerably, resulting in enhanced cycle stability and increased sulfur utilization. Table 1 delineates that many oxides have been effectively functionalized as additives or incorporated into sulfur cathodes, showcasing significant promise for future commercial use in Li–S batteries [15].

Metal oxides typically demonstrate low electrical conductivity, thereby restricting the efficiency of electrochemical reactions that involve anchored polysulfides on their surfaces. For effective utilization, these polysulfides need to diffuse towards neighboring conductive carbon materials. The diffusion of anchored polysulfides across metal oxide surfaces is a critical factor in the design of effective interlayers for sulfur-based batteries. Research [9,28,29] integrating density functional theory (DFT) calculations with experimental validations identifies MgO as a suitable candidate owing to its robust polysulfide-binding capability and minimal diffusion energy barrier, thereby effectively mitigating the polysulfide shuttle and improving the overall battery performance.

This study aims to develop and investigate a novel S/MgO–carbon composite interlayer to enhance the performance of Li–S batteries by addressing key challenges, particularly the polysulfide shuttle effect, which leads to capacity fading and reduced efficiency. The integration of MgO nanoparticles with multiwall carbon nanotubes (MWCNTs) and Super P carbon black enhances the trapping and reutilization of polysulfides. Structural analyses employing X–ray diffraction (XRD), Raman spectroscopy, Fourier transform infrared (FT–IR) spectroscopy, and scanning electron microscopy (SEM) validated the uniform dispersion of MgO, which effectively confines polysulfides and improves the structural integrity. Electrochemical tests, including galvanostatic charge–discharge (GCD) and cyclic voltammetry (CV), revealed improved rate capability and cycle life, with stable capacity retention (~600 mA h g^−1^ over 200 cycles) and high coulombic efficiency (CE) (~99.5%). These findings demonstrate MgO’s role in enhancing sulfur utilization, mitigating the polysulfide shuttle, and improving the overall battery longevity.

The incorporation of MgO into sulfur cathodes has demonstrated significant improvements in suppressing the polysulfide shuttle effect, leading to enhanced cycling stability and sulfur utilization in Li–S batteries. MgO as a functional additive is expected to endow the cathode with stronger chemical confinement against polysulfide shuttling. In addition to exploring other transition metal oxides and composite materials to enhance battery performance, future research could investigate the application of these improved Li–S batteries in space. The unique environmental conditions of space, such as extreme temperatures, vacuum, and radiation, present challenges for battery technologies, but the high energy density and improved cycling stability of MgO–enhanced Li–S batteries make them attractive candidates for space missions. Optimizing the design to ensure stability under these harsh conditions could enable their use in space exploration, satellites, and lunar or Martian habitats, where long-duration energy storage is critical. Additionally, fine–tuning the particle size and surface modification of MgO may enhance its interaction with polysulfides, providing opportunities to develop next–generation Li–S batteries with even greater capacity retention, energy density, and long–term stability.

## 2. Results and Discussion

### 2.1. Structural Measurements

#### 2.1.1. X-Ray Diffraction (XRD)

**Figure 2:**
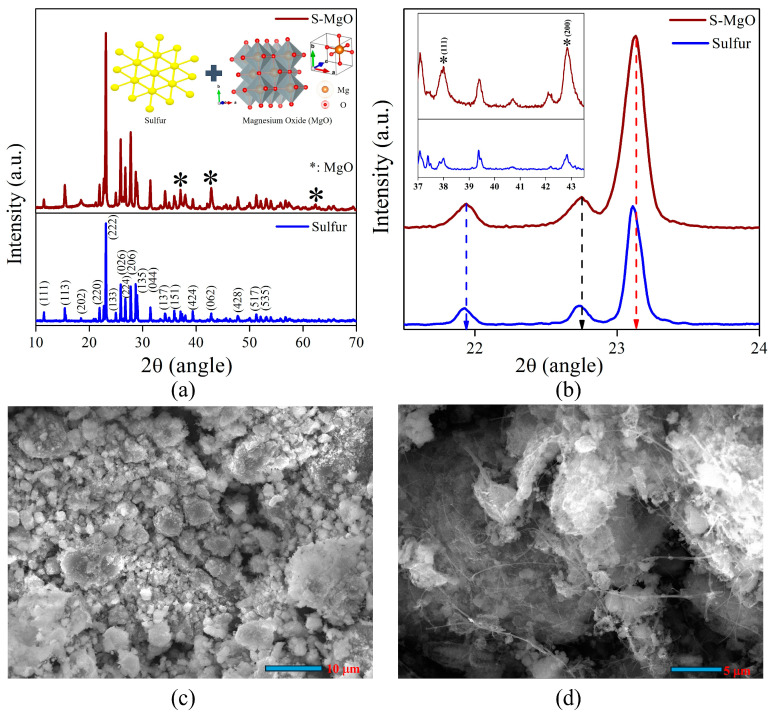
(**a**) XRD patterns of sulfur and S/MgO (inset: Vesta images of sulfur and MgO), (**b**) minimal peak shifting (inset: peaks of MgO), (**c**) SEM images of S/MgO, and (**d**) S/MgO–MWCNTs–Super P composite material.

The XRD patterns of pristine sulfur (S) and S/MgO are shown in Figure 2a. The sulfur patterns exhibit sharp, well-defined peaks, specifically prominent at about 23.11°, 25.81°, and 27.70°, which correspond to (222), (026), and (206), respectively. These are the characteristic peaks of the orthorhombic crystal structure of sulfur (S_8_) [30]. We observed many other reflections, indicated with (hkl) values in Figure 2a. These peaks signify the high crystalline nature and phase purity of sulfur (ICDD/JCPDS reference file 01–07–1888). These peaks remain prominent in the S/MgO composite sample, confirming that sulfur retains its crystallinity after the incorporation of MgO nanoparticles. In addition to sulfur’s peaks, the prepared sample shows distinct peaks at 36.92°, 42.91°, and 63.28° (inset: Figure 2b), attributed to the cubic phase of MgO (ICDD/JCPDS reference file 00–043–1022) [31,32], confirming the successful incorporation into the composite. We observed minimal peak shifting (Figure 2b) to a higher angle in the S/MgO sample (222 peak from 23.11° to 23.15°) (Figure S2), suggesting minor lattice distortion, which might be due to the interaction between sulfur and MgO. Thus, the intensity of the S/MgO prepared (Figure S1) sample is relatively high compared to pristine sulfur, indicating that the presence of MgO nanoparticles enhances the crystallinity of sulfur [33,34]. This leads to MgO acting as a nucleation site, facilitating the crystallization process of sulfur and leading to the stabilization of the sulfur structure. The improved crystallinity and structural stability are beneficial for enhancing the performance of the composite material regarding the application of Li–S batteries [34]. To confirm the phase of the composite materials, we refined the spectra utilizing the HighScore plus software 3.0.5 to analyze the phase, sulfur consisting of the $F_{ddd}$ space group (orthorhombic structure), and MgO as the secondary phase of the $F_{m\bar{3}m}$ space group of the cubic crystal structure, and the refinement maintains the goodness–of–fit factor at less than 3%.

#### 2.1.2. Scanning Electron Microscopy (SEM)

Figure 2c,d shows the SEM images of the S/MgO and S/MgO–MWCNTs–Super P composites, respectively. In the S/MgO composite, the sulfur matrix appears to be uniformly dispersed with the MgO nanoparticles across the surface, preventing sulfur agglomeration and contributing to the overall structural uniformity of the composite, as observed in the XRD analysis. The S/MgO–MWCNTs–Super P composite, however, displays a more complex morphology, with the MWCNTs forming a fibrous network (shown in Figure 2d) intertwined with sulfur and MgO particles, while Super P contributes to a porous, conductive matrix (also shown in Figure S3) [34,35,36]. This interconnected structure enhances electron transport, with the MgO particles remaining well-integrated. The morphology suggests an optimized composite for high conductivity and stability, typically required for Li–S battery applications.

#### 2.1.3. Raman Analysis

**Figure 3:**
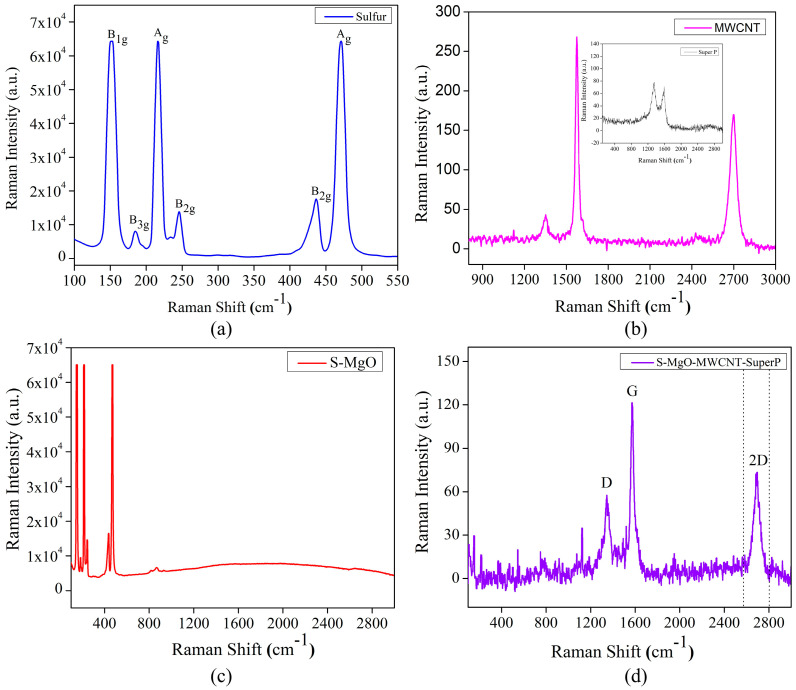
Raman spectra of (**a**) pristine sulfur, (**b**) MWCNT (inset: Super P), (**c**) S/MgO, and (**d**) S/MgO–MWCNTs–Super P.

Figure 3a–d shows the Raman spectra of the pristine sulfur, MWCNT (inset: super P), S/MgO, and S/MgO–MWCNTs–Super P, respectively. However, studies [37,38,39,40] indicated the $D_{4}d$ and $D_{2}h$ [38,41,42,43] point group for the analysis of the Raman spectra of pristine sulfur. For our measurement, we utilized the $D_{2}h$ point group and $F_{ddd}$ space group for α-sulfur of crystalline form. We discussed in detail how the sulfur modes are assigned to specific symmetry in the vibrational mode analysis section of the Supplementary Information (SI). In Figure 3a, there are various Raman active modes, at about 150, 183, 245, 436 cm^−1^, corresponding to non-totally symmetric bending/stretching vibrations with B_1g_, B_2g_, B_3g_ symmetries. Additionally, the modes at 216 and 470 cm^−1^ correspond to symmetric stretching vibrations with A_g_ symmetry [38,41].

Regarding the Raman spectra of the MWCNT and Super P shown in Figure 3b, the MWCNT spectra exhibit characteristic peaks at about 1350 cm^−1^ (D–band), which signify the defects/disorder in the sp^2^ carbon lattice around 1580 cm^−1^ (G–band), suggesting in-plane vibrations of sp^2^–hybridized carbon atoms, and 2700 cm^−1^ (2D band), which is an overtone of the d-band and broader due to the structure of the MWCNT composite [44,45,46]. For the mixture of S/MgO and S/MgO–MWCNTs–Super P, Raman spectra are also shown in Figure 3c,d. In the S/MgO, peaks between 150 cm^−1^ and 500 cm^−1^ predominately show sulfur vibrational active modes. The absence of significant peaks beyond 500 cm^−1^ reflects the lack of observable raman activity for MgO, which typically has a very low Raman cross-section due to its ionic nature and simple vibrational modes that are often raman-inactive or weakly active. Therefore, MgO does not contribute prominently to the spectrum, and the flat signal beyond sulfur’s peaks confirms MgO’s minimal contribution to the raman scattering in this composite. In the mixing of all the precursors, Figure 3d shows characteristic carbon peaks of D, G, and 2D bands at about 1350, 1580, and 2683 cm^−1^, respectively. Significantly, the comparable intensity of the D band to that of the G band in our study serves as a clear indicator of the presence of defects within the examined specimen. The calculated ratio between the intensities of the D and G bands (I_D_/I_G_) is determined to be 0.47, suggesting low–level defects in the sample alongside a higher degree of crystallinity. This observed I_D_/I_G_ ratio provides valuable quantitative insight into the structural characteristics of the examined material, emphasizing the coexistence of defects and a well–defined crystalline structure within the analyzed carbon nanotube sample. This low value of the ratio indicates that MWCNTs retain a substantial degree of crystallinity, even after the incorporation of S and MgO [47,48,49].

#### 2.1.4. Fourier Transform Infrared Spectroscopy

**Figure 4:**
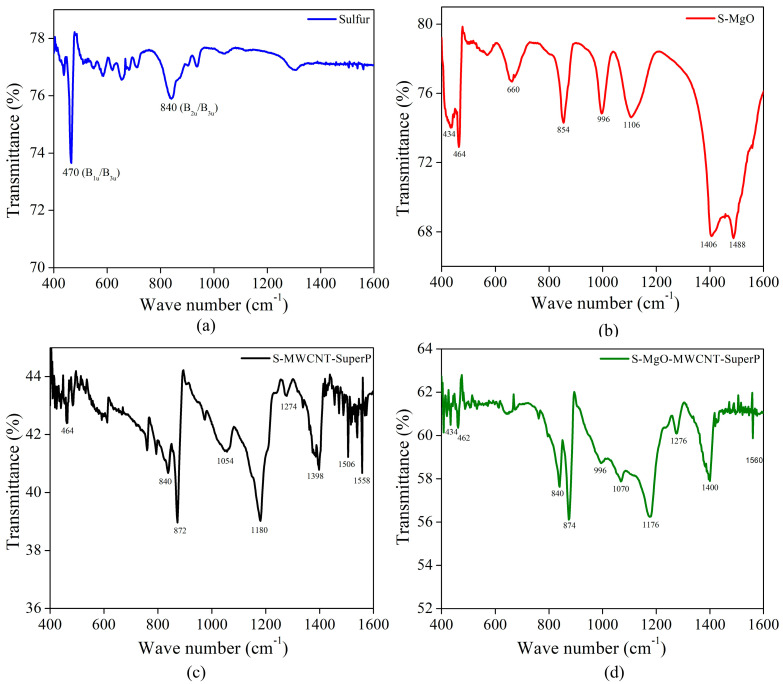
FT–IR spectra of (**a**) pristine sulfur, (**b**) S/MgO, (**c**) S–MWCNT–Super P, and (**d**) S/MgO–MWCNTs–Super P composite.

As we discussed regarding the vibrational mode’s activity in the pristine sulfur (SI section), there are modes B_1u_, B_2u_, and B_3u_, which are IR–active, not Raman-active, and only A_u_ mode, which is neither Raman nor IR active, called silent mode. FT–IR is a well-established spectroscopic technique that is low in cost with simple operation but has yet to be implemented in Li–S batteries under electrochemical evaluation to monitor Li–S batteries. Recent work [50] has established that this technique could be used to detect different LiPS species based on the S–S vibrational mode. Figure 4a shows the spectrum of pristine sulfur, demonstrates the peak about 470 cm^−1^, a characteristic signal of S_8_, and can be attributed to the stretching of S–S bonds, which is more likely consistent with theB_1u_/B_3u_ IR active mode, predicted by group theory for the D_2_h point group [51,52]. Additionally, we observed the other signature mode around 840 cm^−1^, which likely corresponds to the bending mode or deformation mode of the S_8_ ring structure because of the bending, wagging, or twisting modes generally observed in this region. This peak might be attributed to the B_2u_/B_3u_ mode, where sulfur atoms might be undergone out of the plane distortions [42,52,53]. This phenomenon tends to increase the electrical conductivity and catalytic reaction efficiency [54,55]. In Figure 4b, the main peak for identifying Mg=O is observed at 434 cm^−1^ from the stretching bond [56]. The peaks at 1488 cm^−1^ and 1406 cm^−1^ are the bending vibrations of the water molecules due to the humidity in the S/MgO formation process since they are rapidly chemisorbed on the surface of the MgO, as well as the CO_2_ bound to a sulfur [57,58]. Additionally, the other characteristic peaks of sulfur are observed at 464 cm^−1^ and 854 cm^−1^, and the interactions between sulfur and the MgO molecule in the formation of sulfites and sulfates are shown in the peaks at 1106 and 996 cm^−1^, respectively [59]. However, with the MgO composite electrodes, it is found that there are two key absorption regions that provide insightful redox information: the S–S vibrational modes approaching the far–IR region (~470 cm^−1^) and the symmetric SO_3_ stretch vibrational mode of the triflate anion (~854 cm^−1^). Both regions show cyclic evolution during reactions, indicating the disappearance of polysulfides and changes in the triflate ion coordination state, respectively.

In the case of S–MWCNT–Super P, the spectrum indicates new chemical ligands together with sulfur. We observed the characteristic peaks of sulfur at 840 cm^−1^ and 464 cm^−1^; this last peak was displaced by the S–C interactions; in addition, there is the presence of the S–OR (ester group) that can be identified in the peak at 878 cm^−1^ [55]. The peaks shown are associated with C–S, C–C, and C=C bonds, as indicated in the peaks at 1054, 1274, and 1398 cm^−1^ [55], which include the stretching of the sulfate and sulfonated groups, and the peaks at 1550 and 1506 cm^−1^ would be the stretching vibrations of the C–C and C=C bonds [60] characteristic of MWCNTs. Moreover. Figure 4d shows the S/MgO and mixing of the MWCNT/Super P combination. The structure reveals the combination of the signals, where we can observe the presence of the new material in the interaction, the S ions with peaks at 840 and 434 cm^−1^, the characteristic signal of MgO at 434 cm^−1^, and the C=C and C–C interactions at 1560 cm^−1^ and 1400 cm^−1^, respectively.

The formation of the structures between the S-MWCNT and S-MgO is a benefit regarding the use of electrodes for Li–S batteries because both structures can prevent the formation of polysulfides in the cell electrolyte, improving the conductivity and structural stability during multiple charges and discharges. The combination could improve the overall properties, such as improved structural stability for increased electrical conductivity and better containment of the polysulfide formation within the battery electrolyte, resulting in higher energy density.

### 2.2. Electrochemistry

In Li–S batteries, electrochemical measurements play a crucial role in understanding the mechanisms of sulfur redox reactions, polysulfide formation, and overall battery performance. The electrochemical behavior of the sulfur in the battery is characterized by a series of phase transitions and chemical reactions as lithium ions (Li^+^) interact with the sulfur to form different polysulfide intermediates. Figure 1 also highlights the well–known electrochemical reactions involved in the Li–S batteries. Electrochemical techniques such as galvanostatic charge–discharge (GCD) cycling, cyclic voltammetry (CV), and electrochemical impedance spectroscopy (EIS) provide insight into the kinetics of these redox processes, battery capacity, and stability over time.

**Figure 5:**
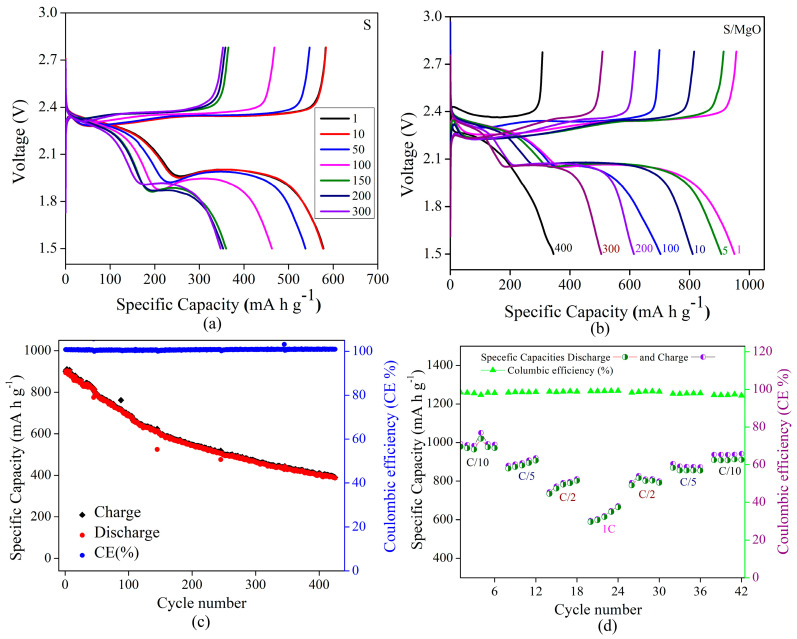
Galvanostatic charge–discharge profile: (**a**) S cathode (without MgO), (**b**) S/MgO composite cathode and (**c**) discharge–specific capacities of S/MgO composite cathode, and (**d**) rate performance of S/MgO composite cathode in Li–S coin cell.

Figure 5 shows GCD profiles of the assembled coin cells of (a) S/MWCNT/Super P/PVDF and (b) S-MgO/MWCNTs/Super P/PVDF, highlighting the characteristic two plateau discharge behavior evident of the typical behavior of Li–S batteries. The first plateau observed at 2.3 V corresponds to the reduction of elemental sulfur (S_8_) to long–chain polysulfides (Li_2_S_6_ to Li_2_S_4_), while the second plateau around 2.1 V is associated with further reduction to insoluble Li_2_S_2_/Li_2_S, consistent with the reported literature on sulfur electrochemistry in Li–S cells [61,62,63]. The cell demonstrated an initial discharge capacity of 950 mA h g^−1^ while incorporating MgO other than the pristine sulfur (600 mA h g^−1^ initial cycles at 0.1 C rate), demonstrating more stable configurations over 400 cycles. The cycling performance and rate of the battery depend on many parameters, like the used electrolyte and its quantity, the active material’s stability and compatibility with the current collector, the applied current for charge and discharge, etc. In the present manuscript, the main focus was to control the polysulfide formation in the Li–S battery via the encapsulation engineering of sulfur with the MgO additive regarding the cathode. However, capacity retention was observed with various C rates. In the S/MgO composite, we still observed the two–stage plateau, but the MgO additive played a crucial role in mitigating the polysulfide shuttle effect by chemically absorbing polysulfides and reducing the dissolution into electrolytes. This interaction between the MgO and polysulfides prevents the loss of active sulfur material and enhances the utilization, consistent with the state of the art [34,64,65]. Furthermore, the fabricated cell exhibited a stable nature, as evidenced by the CE consistently exceeding 99.5%, depicted in Figure 5c. Figure 5d shows the cyclic performance and respective CE of the assembled coin cell, demonstrating the impact of varying C rates (C/10 to 1 C) on the electrochemical behavior. We observed a specific discharge capacity ≅1000 mA h g^−1^ at a lower C/10 rate, which retained its capacity ~650 mA h g^−1^ at 1 C upon increasing the current and resumed the pattern of the original capacity while decreasing the current. This natural phenomenon of batteries might be due to kinetic limitations and incomplete redox reactions as high current densities reduce the time for full sulfur conversion and lead to lower specific capacities [4,47,49,66,67]. Moreover, the recovery of the specific capacity occurs upon returning to lower C values, which suggests a robust structure of the composite, enabling reversible sulfur reactions and stable performance over extended cycles (Figure S5) [68]. Notably, CE remains at about 99.5% throughout all the cycles, signifying highly reversible electrochemical reactions and minimal loss of active material, supported by the polysulfide–trapping capability of MgO, which prevents dissolution and enhances the battery performance. The S/MgO–MWCNT–Super P composite demonstrates significant improvements in sulfur retention and cycling stability compared to earlier oxide–based composites. Oxides like TiO_2_, CeO_2_, and Fe_3_O_4_ (Table 1) have demonstrated effectiveness in enhancing sulfur cathode performance. In contrast, the current study achieves a stable discharge capacity of approximately 600 mA h g^−1^ over 200 cycles at 0.1 C and maintains around 650 mA h g^−1^ at 1 C, alongside a high CE of ~99.5%. The results demonstrate comparability to, and in certain instances surpass, the performance of the established oxide composites, such as TiO_2_/S (850 mA h g^−1^ at 0.5 C), CeO_2_/S (611 mA h g^−1^ at 0.5 C), and Fe_3_O_4_/S (610 mA h g^−1^ at 1 C). The S/MgO–MWCNT–Super P composite demonstrates superior rate capability, high stability, and improved sulfur utilization, highlighting its potential as a viable candidate for the advancement of Li–S battery technologies, especially in applications that demand both high energy density and long-term stability.

**Figure 6:**
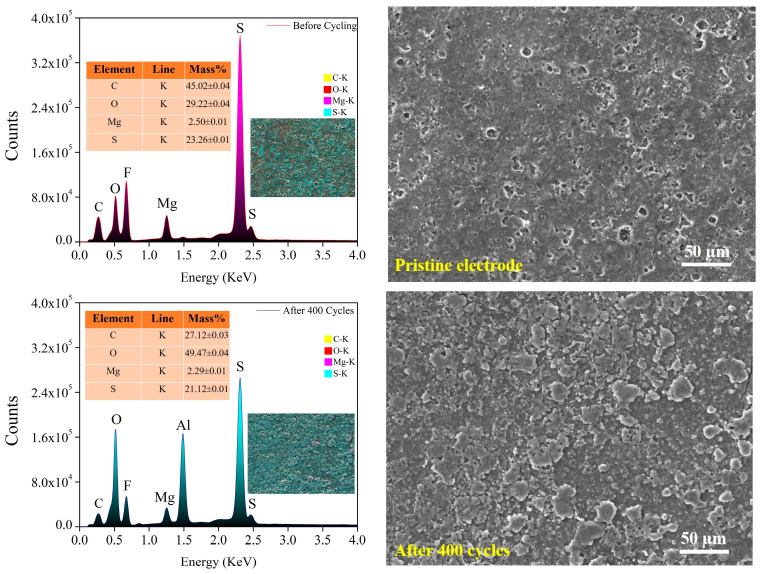
SEM–EDS images of pristine and after 400 cycles of S/MgO cathode.

After the cycling of the cell, we conducted the investigation of the EDS and SEM (Figure 6) of the S/MgO composite electrode for uncycled and after 400 cycles, indicating significant structural and compositional alterations that highlight the electrode’s stability. In the pristine electrode, MgO nanoparticles were found to be randomly and sparsely distributed on the surface of the micrometer–sized sulfur particles (Figure S4). Remarkably, the electrodes remained intact even after 400 cycles, a notable achievement considering that sulfur electrodes typically face challenges in sustaining extended cycles without additives [34,64]. Notably, there is no evidence of fracture observed in the pristine sample, whereas some signature of fracture becomes apparent after the completion of 400 cycles, underscoring the protective role of MgO nanoparticles in maintaining the structural integrity of the electrode during the cycling process. From the EDS analysis of the cycled cathode, it shows a reduction in the sulfur content from 23% to 21%, suggesting sulfur loss, likely attributed to the dissolution of polysulfides into the electrolyte, a prevalent issue in Li–S batteries that contributes to loss of active material and capacity degradation over prolonged cycles. The increase in the oxygen content indicates a possible interaction between MgO and polysulfides, which may lead to the formation of sulfur–oxygen complexes or other oxygen containing byproducts [69,70,71]. The interaction between MgO and polysulfides is influenced by MgO’s significant chemical affinity for polysulfides, which aids in confining them within the cathode matrix, thus reducing their migration—an essential factor for improving the long–term electrochemical stability of Li–S batteries. The results highlight the dual role of MgO as a conductive matrix and chemical adsorbent for polysulfides, enhancing the sulfur retention and cycling performance. Furthermore, the capacity of MgO to capture polysulfides may lead to an enhanced oxygen signature detected in EDS analysis as increased formation of oxidized species is probable during the cycling process. This behavior reinforces the role of MgO in stabilizing the sulfur cathode and mitigating capacity fade.

**Figure 7:**
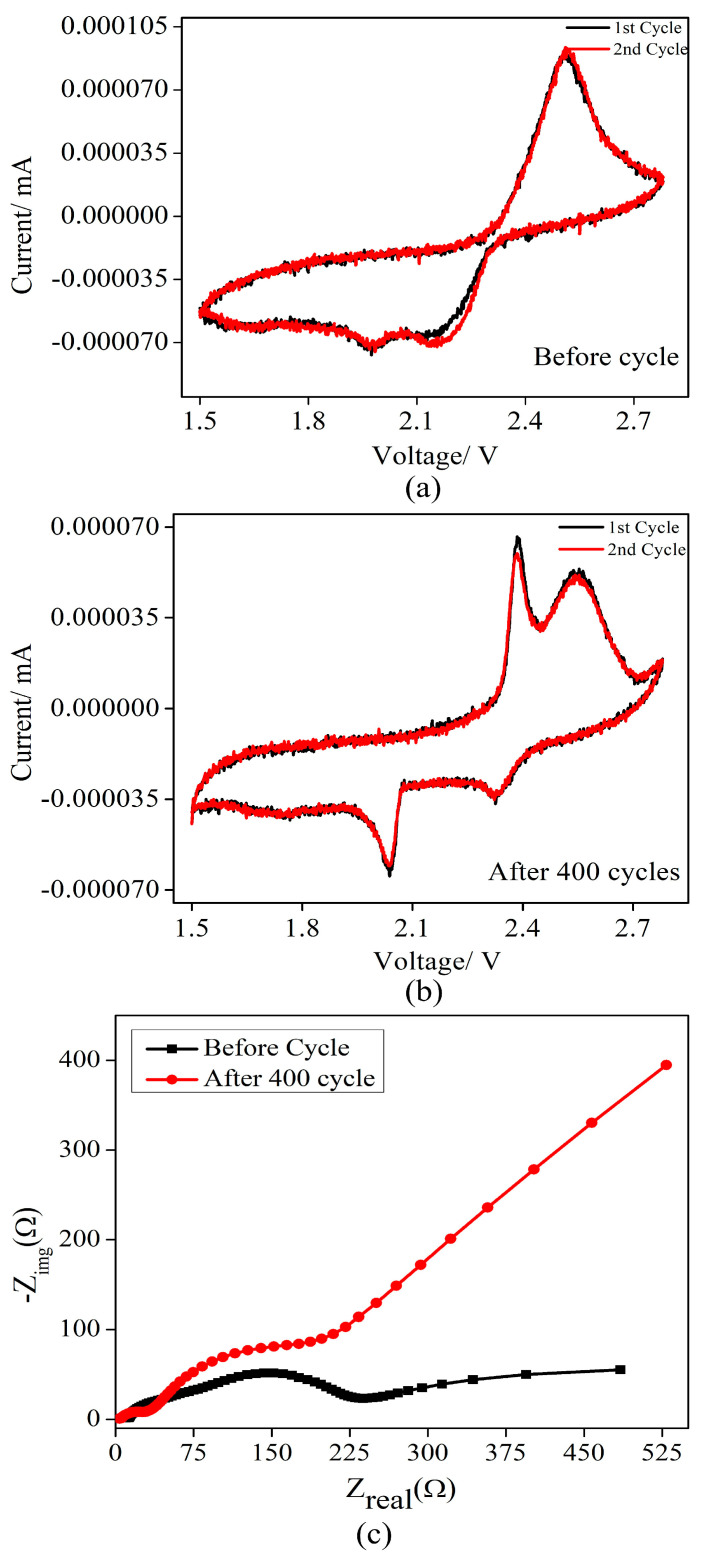
Cyclic voltammetry (CV) of (**a**) uncycled cathode, (**b**) after 400 cycles, and (**c**) EIS performance of S–MgO.

The cyclic voltammetry (CV) profiles (Figure 7a,b) further confirm these findings, showing stable redox activity of sulfur before and after 400 cycles, with MgO effectively trapping polysulfides and maintaining electrode conductivity. The higher current density after 400 cycles suggests improved ionic and electronic pathways provided by MWCNT and Super P. The electrochemical characteristics of a pristine electrode, as reported in the literature [66,72,73], reveal two reduction peaks at 1.95 V and 2.2 V, along with a single oxidation peak at 2.45 V, indicative of the typical sulfur reduction and oxidation processes. The subsequent investigation of the CV characteristics after 400 cycles, within the operating voltage range of 1.5–2.78 V, shows a stable electrochemical performance regarding the reduction. The essentially coincident CV curves after the first cycle suggest sustained stability. Moreover, the observed shifting in the peaks implies alterations in the thermodynamics of the sulfur reduction and Li–S oxidation, hinting at potential improvements in energy barriers. The two reduction peaks are associated with sulfur reduction reactions, progressing from sulfur to lithium polysulfides (Li_2_S_n_, 4 < *n* < 8), and further reduction to Li_2_S_2_/Li_2_S. The oxidation peak corresponds to the reaction from Li_2_S to lithium polysulfides. The stability regarding the oxidation and reduction peaks indicates that the S–MgO composite electrode Li–S cell exhibits significant electrochemical performance, reinforcing its suitability for practical applications [9,21,26,27,73]. Electrochemical impedance spectroscopy (EIS) (Figure 7c) further confirmed the enhanced performance, with a decrease in solution resistance (from 11.77 Ω to 3.9 Ω) and charge transfer resistance (from 185 Ω to 156 Ω), signifying improved ionic conductivity and efficient charge transfer [74]. In the process of the electrochemical reaction in electrodes, the Li–ion distributions at the surface and inside the electrode differ, which manifests as a Li–ion concentration gradient and drives Li–ion diffusion. In most cases, Li diffusion plays an import role in the kinetic process that occurs in the electrode materials because the diffusion determines the reaction velocity of electrode materials and thus the rate performance of the electrode. However, the increased diffusion impedance at low frequencies points to diffusion limitations, likely due to polysulfide accumulation or solid electrolyte interphase (SEI) growth after extended cycling. These findings reinforce the suitability of the S–MgO composite for long-term Li–S battery applications as it effectively mitigates the shuttle effect and enhances sulfur utilization [59,63,73].

## 3. Materials and Methods

### 3.1. Materials

We utilized the following materials in our study, sourced from Sigma-Aldrich unless otherwise noted: magnesium oxide (MgO, nanopowder, ≤50 nm particle size), Super-P carbon black, sulfur (S, 99.5–100.5%), polyvinylidene fluoride (PVDF, Mw 1000–1200 kg mol^−1^, Solef 5130, Solvay, Brussels, Belgium), 1,3-dioxolane (DOL, 99%), 1,2-dimethoxyethane (DME, 99.5%), bis(trifluoromethane) sulfonamide lithium (LiTFSI, 99.95% trace metals basis), and lithium nitrate (LiNO_3_, 99.99%, trace metals basis). Additionally, the polymer-based separator used for coating was polyphenylene ether (PPE, Celgard Chemicals, Charlotte, NC, USA). All materials were used as received, without further purification. We would like to clarify that the mention of company names is solely for the purpose of accurately describing the materials/ instruments used in our research and does not imply any form of advertisement or endorsement. 

### 3.2. Cathode Preparation, Electrolyte Preparation, and Li–S Coin Cell Assembly

**Figure 8:**
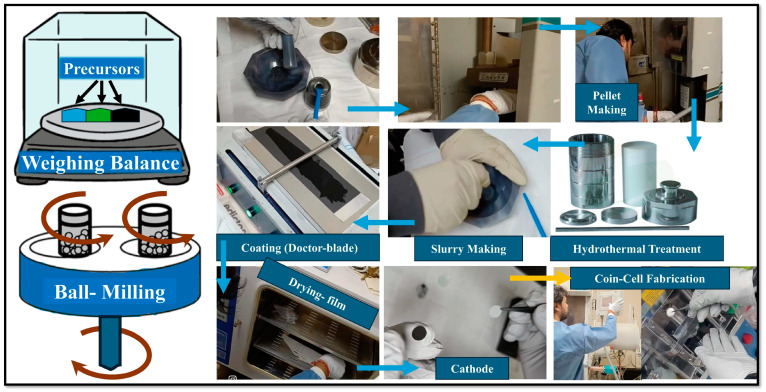
Schematics of cathode preparation.

In the cathode, compositions are represented as (S_0.9_MgO_0.1_)_0.6_ + (MWCNT)_0.2_ + (Super P)_0.1_ + (PVDF)_0.1_. Figure 8 illustrates the approach for cathode preparation, wherein we initially combine 90% sulfur with 10% MgO using ball milling. Subsequently, 60% of this mixture was amalgamated with 20% MWCNT and formed into pellets for further processing by the heat diffusion technique in the autoclave and kept in an oven at 150 °C for 6 h. Subsequently, during the preparation of the slurry, 10% Super P and 10% PVDF (solution with NMP) were included, and the combination was crushed in an agate mortar until homogenous. To attain the optimal viscosity for electrode preparation, the addition of 1–2 mL of NMP is advised. Additionally, we prepared the pristine sulfur cathode as (S)_0.6_ + (MWCNT)_0.2_ + (Super P)_0.1_ + (PVDF)_0.1_. All components were stirred together in a slurry maker until a homogeneous slurry was obtained. The slurry was evenly spread over an aluminum sheet using a doctor blade machine, then dried at 60 °C for 16 h in a vacuum oven. A die cutter with a 10 mm diameter (MTI Corporation, Richmond, CA, USA) was used to punch the electrodes, and their weight was measured. The electrodes were then transferred into an Ar–filled glove box (MBraun, Garching bei München, Germany) for coin cell assembly. The active mass loading of the final electrodes was approximately 1.1–1.4 mg cm^–2^, and cell was crimped utilizing MTI’s Digital Pressure Controlled Electric Crimper for CR20XX Coin Cells (Ar Glovebox Compatible) MSK-160E (Richmond, CA, USA) and pressure were maintained from 0.831–0.835 T.

#### Electrolyte Preparation and Assembly of Coin Cell 2032

For efficient trapping of polysulfides, a 1 M solution of bis(trifluoromethane) sulfonamide lithium (LiTFSI) in a solvent mixture of 1,3-dioxolane (DOL) and 1,2-dimethoxyethane (DME) in a 1:1 ratio was employed as the electrolyte. Initially, dried the LiTFSI and lithium nitrite (LiNO_3_) overnight at 65–70°C to remove the possible moisture. Subsequently, 2 wt% of lithium nitrite (LiNO_3_) was added to enhance the electrolyte’s performance. The fixed amount of electrolyte in various coin cells was maintained at 11–12 μL mg^–1^ of active sulfur material. This tailored electrolyte composition was designed to optimize the performance of the Li–S battery system in the experimental setup. Coin cells were assembled using CR2032 components sourced from Landt Instrument, including a cathode cap, anode cap, spring, and spacer. The setup also included a polypropylene (PP) separator (Celgard 2400, 16 mm), LiTFSI electrolyte, and Li chips (0.6 mm thick, supplied by MSE). A Li chip was employed as the counter electrode (anode) in the half-cell configuration for Li–S batteries. The coin cell assembly steps are shown in Figure S6.

### 3.3. Characterization

The crystal structure was analyzed using an X-ray diffractometer (XRD, Rigaku Smart Lab, Tokyo, Japan) with CuK_a_ radiation and a wavelength (λ) of 1.5405 Å. The instrument was configured in Bragg–Brentano (θ–2θ) geometry, scan rate 0.01, 2θ 10–70°, operated at 40 kV and 44 mA, power 1.76 kW, and measurement error ±0.01. To analyze the data, we utilized Highscore Plus software 3.0.5 for peak matching and phase confirmation, visualized using Vesta. Scanning electron microscopy (SEM) images and energy dispersive X–ray spectroscopy (EDS) spectra were acquired with a JOEL JEM–1400Plus (Peabody, MA, USA) microscope equipped with a LaB6 thermionic source operating at 120 kV. For the analysis of normal modes of vibration, Raman and Fourier transform infrared spectroscopy (FT–IR) spectra were utilized. Raman spectra were recorded from 100 to 3000 cm^−1^ using a Horiba-Jobin T64000 spectrometer (Longjumeau, France) in backscattering geometry, with an excitation wavelength of 514.5 nm (a confocal microscope with an 80× objective with a numerical aperture of 0.9). We utilized the suitable power (1–2 mW on prepared sample) for the prepared sample based on the spectra observation and sample nature. Also, FTIR spectra of samples were recorded by UATR HR Spectrum Two Perking Elmer (Shelton, CT, USA). Data spectra were obtained in the range of 400–4000 cm^−1^ using 32 scans and resolutions of 2 cm^−1^.

Additionally, electrochemical evaluations, including charge–discharge profiles, cycling stability, cyclic voltammetry, and electrochemical impedance spectroscopy (EIS), were conducted to comprehensively assess the battery performance. Galvanostatic charge–discharge (GCD) rate performance curves were obtained using the multi-channel battery test system CT3002A (Landt, Vestal, NY, USA) in a voltage range of 1.5–2.78 V (vs. Li/Li⁺), applying various currents. Cyclic voltammetry (CV) tests were conducted to evaluate the cycling performance and stability of the cathode material using an Arbin instrument at scan rates (0.1 mV/s). Electrochemical impedance spectroscopy (EIS) measurements, conducted before and after cycling at the open circuit voltage (OCV), utilized a signal amplitude of 10 mV within a wide frequency range, using a Gamry Instrument (Interface 1010E Potentiostat/Galvanostat/ZRA, 26081; Warminster, PA, USA).

## 4. Conclusions

This study shows that MgO significantly improves the stability and discharge capacity of Li–S batteries by functioning as a polysulfide–trapping agent. It sequesters dissolved polysulfide intermediates from the sulfur cathode, thereby enabling the active material to persist in electrochemical reactions. Structural and electrochemical analyses, such as XRD, Raman, FT–IR spectroscopy, and cyclic voltammetry, validate the role of MgO in reducing the polysulfide shuttle effect and enhancing sulfur utilization. Incorporating MgO into the sulfur matrix diminishes capacity fading and enhances long–term cycling stability. Following 400 cycles, the S–MgO composite retains its structural integrity, as demonstrated by the SEM and EDS analyses, revealing only slight sulfur loss and an increase in the oxygen content attributable to interactions between sulfur and MgO. The composite demonstrates stable redox behavior and attains a high CE of 99.5%, indicating that MgO serves as an effective additive for enhancing the lifespan and efficiency of Li–S batteries. The results demonstrate that MgO is a promising additive for advancing Li–S battery technologies by enhancing both capacity retention and cycling stability.

## Figures and Tables

**Table 1:** Current state–of–the–art oxide materials for enhancing cathode performance in Li–S batteries.

Oxides as Cathode Additive	Mechanism	Experimental Findings	Reference
Al_2_O_3_/S composite	Adsorbs LiPSs, enhancing cycle performance.	660 mA h g^−1^ over 25th cycle	[16]
TiO_2_/S composite	Traps LiPSs and improves sulfur retention and cycle life	850 mA h g^−1^ (0.5 C, 200 cycles) ~97%	[17]
Mg_0.6_Ni_0.4_O/S composite	Prevents LiPS dissolution, enhancing redox reactions and durability	1185 mA h g^−1^ (50 cycles)	[18]
MCM/Nb_2_O_5_ composite	Adsorbs polysulfides, reducing shuttle effect and improving sulfur retention	650 mA h g^−1^ (2 C, 500 cycles) ~98%	[19]
NiO–CNT/S composite	Polysulfide adsorption reduces capacity fade and enhances cycle stability	609 mA h g^−1^ (0.1 C, 160 cycles) ~96%	[20]
SnO_2_/S composite	Strong polysulfide adsorption, limiting shuttle effect and improving retention	550 mA h g^−1^ (0.1 C, 700 cycles) ~95%	[21]
CeO_2_/S composite	Traps LiPSs, reducing shuttle effect and improving capacity	611 mA h g^−1^ (0.5 C, 200 cycles), 3.5 mg/cm^2^ high loading ~99%	[22]
MoO_2_@CNT/S composite	Polysulfide capture and catalytic conversion, improving sulfur utilization	540 mA h g^−1^ (1 C, 700 cycles) ~97%	[23]
Co_3_O_4_/S composite	Adsorbs and catalyzes polysulfide conversion, reducing capacity fade	694 mA h g^−1^ (0.2 C, 550 cycles) ~98%	[24]
rGO@ZnO/S composite	High surface area for polysulfide trapping, enhancing sulfur retention	674 mA h g^−1^ (1 C, 400 cycles) ~96%	[25]
Fe_3_O_4_/S composite	Strong polysulfide adsorption, reducing shuttle effect and stabilizing sulfur	610 mA h g^−1^ (1 C, 1000 cycles) ~98%	[26]
MnO_2_@rGO/S composite	Traps and catalyzes polysulfide conversion, improving cycle stability	578 mA h g^−1^ (0.2 C, 100 cycles) ~95%	[27]
S/MgO–MWCNT–Super P	Traps LiPSs, reducing shuttle effect and improving reversibility	~600 mA h g^−1^ over 200 cycles at 0.1 C, ~650 mA h g^−1^ at 1 C; ~99.5%	Present study

## Data Availability

The data presented in this study is available on request from the corresponding author.

## Supplementary Materials

The following supporting information can be downloaded at: https://www.mdpi.com/article/10.3390/molecules29215116/s1, the supplementary materials include the following tables and figures: Tables: Table S1 (Standard character table for $D_{2}h$ point group symmetry), Table S2 (Total characters representation in $D_{2}h$ point group), Table S3 (Translational characters in $D_{2}h$ point group), Table S4 (Rotational characters in $D_{2}h$ point group), and Table S5 (Vibrational characters in $D_{2}h$ point group). Figures: Figure S1 (XRD spectra (2q vs intensity) of (a) pristine sulfur and (b) S/MgO), Figure S2 (Peak shifting of 222 peak around 23 degrees), Figure S3 (SEM images of sulfur and composites), Figure S4 (SEM image of S-MgO), Figure S5 (Rate performance of Li–S coin cell with continuous cycling), and Figure S6 (Assembly of 2032 coin cell for Li–S batteries).
