# Correction: Choudhary et al. Encapsulation Engineering of Sulfur into Magnesium Oxide for High Energy Density Li–S Batteries. *Molecules* 2024, *29*, 5116

**DOI:** 10.3390/molecules30030707

**Published:** 2025-02-05

**Authors:** Sunny Choudhary, Nischal Oli, Shweta Shweta, Satyam Kumar, Mohan K. Bhattarai, Carlos Alberto Malca-Reyes, Rajesh K. Katiyar, Balram Tripathi, Liz M. Díaz-Vázquez, Gerardo Morell, Ram S. Katiyar

**Affiliations:** 1 Department of Physics, University of Puerto Rico at Río Piedras, San Juan, PR 00931, USA; 2 Department of Chemistry, University of Puerto Rico at Río Piedras, San Juan, PR 00931, USA; 3 Department of Physics, S.S. Jain Subodh P.G. (Autonomous) College, Jaipur Rajasthan 302004, India

In the original publication [1], there was a mistake in Figure 3a and its interpretation. The errors occurred due to the assignment of mode was not properly ordered. To support this correction, Reference [41] was replaced with a new reference.

The corrected text and Figure 3 appear below.

In Figure 3a, there are various Raman active modes, at about 150, 183, 245, 436 cm^−1^, corresponding to non-totally symmetric bending/stretching vibrations with B_1g_, B_2g_, B_3g_ symmetries. Additionally, the modes at 216 and 470 cm^−1^ correspond to symmetric stretching vibrations with A_g_ symmetry [38,41].

The replaced Reference [41] appears below.

Wu, H.L.; Huff, L.A.; Gewirth, A.A. In situ Raman spectroscopy of sulfur speciation in lithium–sulfur batteries. *ACS Appl. Mater. Interfaces* **2015**, *7*, 1709–1719. https://doi.org/10.1021/am5072942.

The authors state that the scientific conclusions are unaffected. This correction was approved by the Academic Editor. The original publication has also been updated.

## Figures and Tables

**Figure 3:**
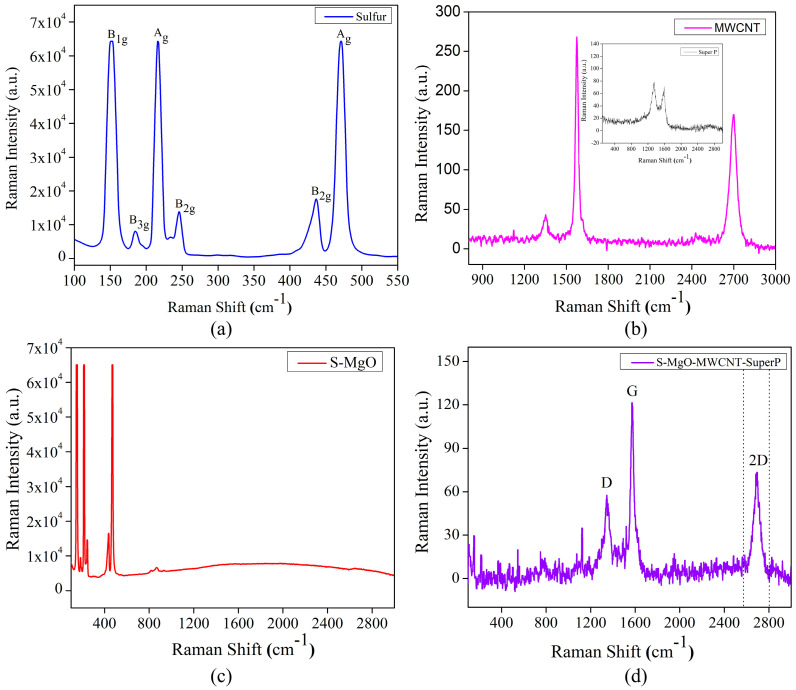
Raman spectra of (**a**) pristine sulfur, (**b**) MWCNT (inset: Super P), (**c**) S/MgO, and (**d**) S/MgO–MWCNTs–Super P.
