# Supplementary material for: Encapsulation Engineering of Sulfur into Magnesium Oxide for High Energy Density Li–S Batteries
Source: Molecules. 2024 Oct 30;29(21):5116. doi: 10.3390/molecules29215116 (PMC11547636; doi:10.3390/molecules29215116)
Supplement: Supplementary file 1 [file molecules-29-05116-s001.zip › molecules-3267708-supplementary.pdf]

Supplementary Materials

Encapsulation Engineering of Sulfur into Magnesium Oxide for High Energy Density Li-S Batteries

Sunny Choudhary[1*], Nischal Oli[1], Shweta Shweta[1], Satyam Kumar[1], Mohan K. Bhattarai[1], Carlos Alberto Malca-Reyes[2], Rajesh K. Katiyar[1], Balram Tripathi[1,3], Liz M. Diaz-Vazquez[2], Gerardo Morell[1], **Ram S Katiyar**[1*]

1) Department of Physics, University of Puerto Rico at Río Piedras, San Juan, PR 00931, United States.
2) Department of Chemistry, University of Puerto Rico at Río Piedras, San Juan, PR 00931, United States
3) Department of Physics, S S Jain Subodh P.G. (Autonomous) College, Jaipur Rajasthan 302004, India.

* Correspondence: souryabhardwaj@gmail.com (S.C.); ram.katiyar@upr.edu (R.S.K.)

Vibrational Modes of Sulfur: Sulfur in its crystalline form, particularly in the F_{ddd} space group, displays unique vibrational characteristics due to its eight-membered ring structure and high symmetry in the crystal lattice. The vibrational analysis of sulfur is critical for understanding its physical and chemical behavior, particularly in sulfur-based materials. In this study, we utilized Raman spectroscopy to investigate the vibrational modes of crystalline sulfur and assigned the observed bands based on the **D_2h** point group symmetry, which corresponds to the orthorhombic crystal structure of sulfur in the F_{ddd} space group.

Raman spectra were recorded for crystalline sulfur, and the vibrational modes were analyzed using group theory based on the **D_2h** symmetry of the sulfur molecule in the F_{ddd} structure. The normal modes of vibration were assigned by decomposing the vibrational degrees of freedom into irreducible representations according to the **D_2h** character table.

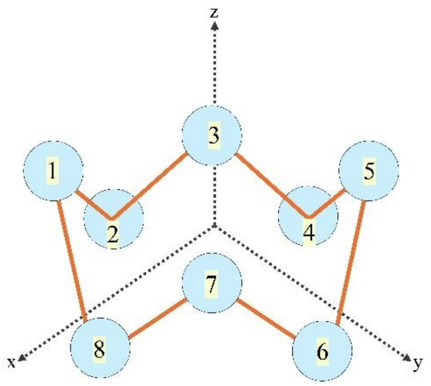

In the case of crystalline sulfur, there are 8 sulfur atoms in the unit cell

Total degree of freedom=3N = 3 * 8 = 24 (3T 3R 18V)

Utilizing this information, we will analyze the normal modes of vibrations and interpret our experimental data.

***Table S1** Standard character table for D_2h point group symmetry*

D_2h	E	$C_2(z)$	$C_2(y)$	$C_2(x)$	i	σ(xy)	σ(xz)	σ(zx)	Basis Functions / Mode Types
A_g	1	1	1	1	1	1	1	1	Symmetric stretching (z^2, x^2, y^2)
B_{1g}	1	1	-1	-1	1	1	-1	-1	In-Plane Symmetric Twisting/ Bending (x^2-y^2)
B_{2g}	1	-1	1	-1	1	-1	1	-1	Symmetric out-of-plane bending (xz)
B_{3g}	1	-1	-1	1	1	-1	-1	1	Symmetric torsional modes (yz)
A_u	1	-1	1	1	-1	-1	-1	-1	Antisymmetric out-of-plane motions
B_{1u}	1	1	-1	-1	-1	-1	1	1	Antisymmetric stretching (Rx)

B_{2u}	1	-1	1	-1	-1	1	-1	1	Antisymmetric out-of-plane deformation (Ry)
B_{3u}	1	-1	-1	1	-1	1	1	-1	Antisymmetric torsional modes (Rz)

Where E: Identity operation; C_2(z/y/x) (180° rotations around the z, y, and x axes, respectively.)

i (Inversion (center of symmetry)); σ(xy), σ(xz), σ(yz) (Reflections in the respective planes.)

<u>Characters (χ) for each symmetry operation in the reducible representation Γ_{vib}</u>

Construct the Total Reducible Representation (Γ_{total})

We use the 3N Cartesian displacement coordinates (x, y, z for each atom) as basic functions.

Symmetry Operations and Characters

Since all symmetry operations in D_2h are unique (each occurs once), we calculate the character ($\boldsymbol{\chi_{total}}$) for each operation based on how the atomic displacement vectors transform.

1. Identity (E): All atoms remain in place and Each atom contributes 3 (x, y, z displacements).

$$\chi_{total}(E) = 8\ atoms\ \times 3 = 24 \quad (1)$$

2. C_2 Rotations and Inversion (C_2 (z), C_2 (y), C_2 (x), i): Atoms are moved to new positions; no atoms remain fixed. Hence, contribution to the character is zero.

$$\chi_{total}(\mathrm{C}_2\ or\ i) = 0 \quad (2)$$

3. Mirror Planes (σ(xy), σ(xz), σ(yz)): Some atoms lie on the mirror planes and remain fixed. For each fixed atom: Displacements in the plane: +1 and Displacements perpendicular to the plane: -1. Assuming the following atoms lie on the mirror planes:

- **σ(xy):** Atoms 1 and 5
- **σ(xz):** Atoms 3 and 7
- **σ(yz):** Atoms 2 and 6

Calculating Characters:

$$\boldsymbol{\chi_{total}(\sigma(\mathrm{xy})) = 2 \times (1 + 1 - 1) = 2} \quad (3)$$

$$\boldsymbol{\chi_{total}(\sigma(\mathrm{xy})) = 2 \times (1 - 1 + 1) = 2} \quad (4)$$

$$\boldsymbol{\chi_{total}(\sigma(\mathrm{xy})) = 2 \times (-1 + 1 + 1) = 2} \quad (5)$$

Table S2 Total characters representation in D_2h point group

Operations	χ_{total}
**E**	24
**C_2(z)**	0
**C_2(y)**	0
**C_2(x)**	0
**i**	0
**σ(xy)**	2
**σ(xz)**	2
**σ(yz)**	2

Translational Representation ($\Gamma_{translational}$): Basis Functions: x (B_{3u}), y (B_{2u}), z (B_{1u})

Table S3 Translational characters representation in D_2h point group

Operations	x (B_{3u})	y (B_{2u})	z (B_{1u})	$\chi_{translational}$
**E**	1	1	1	3
**C_2(z)**	-1	-1	1	-1
**C_2(y)**	-1	1	-1	-1
**C_2(x)**	1	-1	-1	-1
**i**	-1	-1	-1	-3
**σ(xy)**	1	1	-1	1
**σ(xz)**	1	-1	1	1
**σ(yz)**	-1	1	1	1

Rotational Representation ($\Gamma_{rotaional}$): Basis Functions: Rx (B_{3g}), Ry (B_{2g}), Rz (B_{1g})

Table S4 Rotational characters representation in D_2h point group

Operations	Rx (B_{3g})	Ry (B_{2g})	Rz (B_{1g})	$\chi_{translational}$
**E**	1	1	1	3
**C_2(z)**	-1	-1	1	-1
**C_2(y)**	-1	1	-1	-1
**C_2(x)**	1	-1	-1	-1
**i**	1	1	1	3
**σ(xy)**	-1	-1	1	-1
**σ(xz)**	-1	1	-1	-1
**σ(yz)**	1	-1	-1	-1

Table S5 *Vibrational characters representation in D_2h point group*

**Operations**	$\chi_{vibrations} = \chi_{total} - \chi_{translational} - \chi_{vibrations}$
**E**	18
**C_2(z)**	2
**C_2(y)**	2
**C_2(x)**	2
**i**	0
**σ(xy)**	2
**σ(xz)**	2
**σ(yz)**	2

Decompose $\Gamma_{vibrations}$ into Irreducible Representations

We use the reduction formula:

$$a_i = \frac{1}{h}\sum_{R} \chi_{vibrations}(\boldsymbol{R}) \times \chi_i(\boldsymbol{R}) \times \boldsymbol{n_R} \qquad (6)$$

- $\boldsymbol{a_i}$**:** Coefficient for each irreducible representation
- **h:** Order of the group (8 for D_{2h}).
- $\boldsymbol{n_R}$**:** Number of operations in each class (1 for D_{2h}).

$$\boldsymbol{\Gamma_{vibrations} = 4\,A_g + 2B_{1g} + 2B_{2g} + 2B_{1g} + 2A_u + 2B_{1u} + 2B_{2u} + 2B_{1u}} \qquad (7)$$

Raman-Active Modes: Sulfur

- Symmetric Representations:
  - A_g
  - B_{1g}
  - B_{2g}
  - B_{3g}

Infrared-Active Modes

- Representations Transforming Like x, y, z:
  - B_{1u}
  - B_{2u}
  - B_{3u}

Silent mode: A_u

Sulfur octamer (S_8), in its crown-shaped conformation, belongs to the D_2h point group and exhibits 18 vibrational modes. The $\mathbf{A_g}$, $\mathbf{B_{1g}}$, $\mathbf{B_{2g}}$, $\mathbf{B_{3g}}$ modes are Raman-active, as they are symmetric under inversion and specific rotational symmetries, making them observable in Raman spectroscopy. In contrast, the $\mathbf{B_{1u}}$, $\mathbf{B_{2u}}$, $\mathbf{B_{3u}}$ modes are infrared-active due to their antisymmetric nature under inversion and certain mirror plane operations, allowing detection through infrared spectroscopy. A_u is neither Raman active nor IR active, called silent mode [1–5]. This symmetrical analysis of S_8 provides crucial insights into its vibrational characteristics, facilitating the interpretation of its spectroscopic behavior in both infrared and Raman spectra, which is discussed in the manuscript.

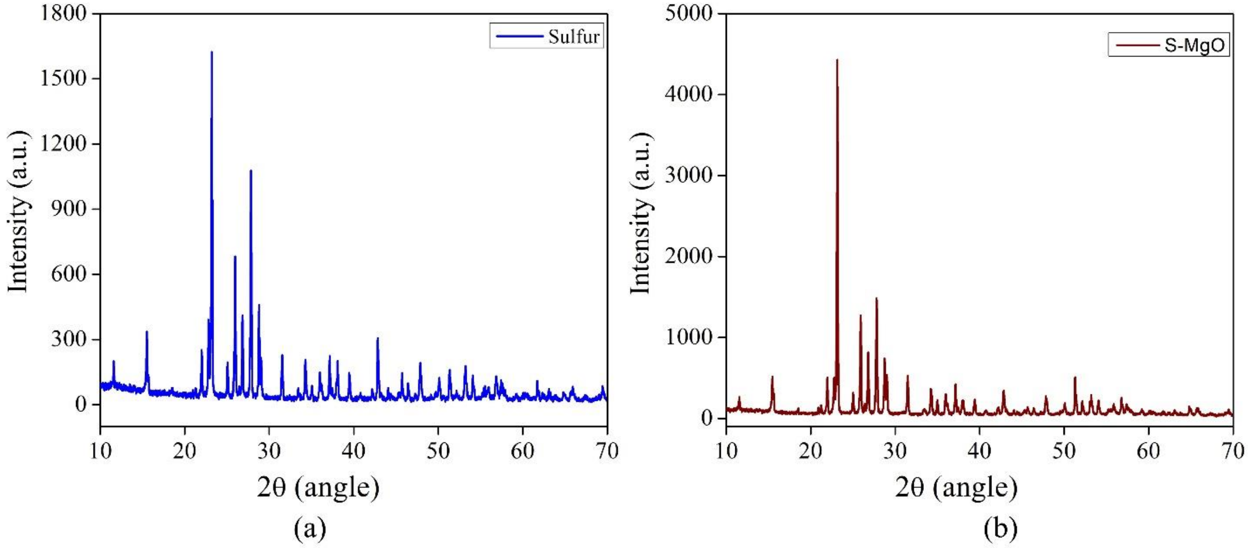

Figure S1 *XRD spectra (2θ vs Intensity) of (a) Pristine sulfur and (b) S/MgO*

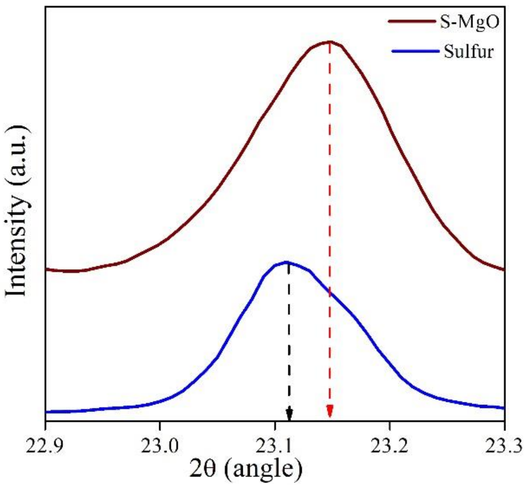

Figure S2 *Peak shifting of 222 peak around 23 degree*

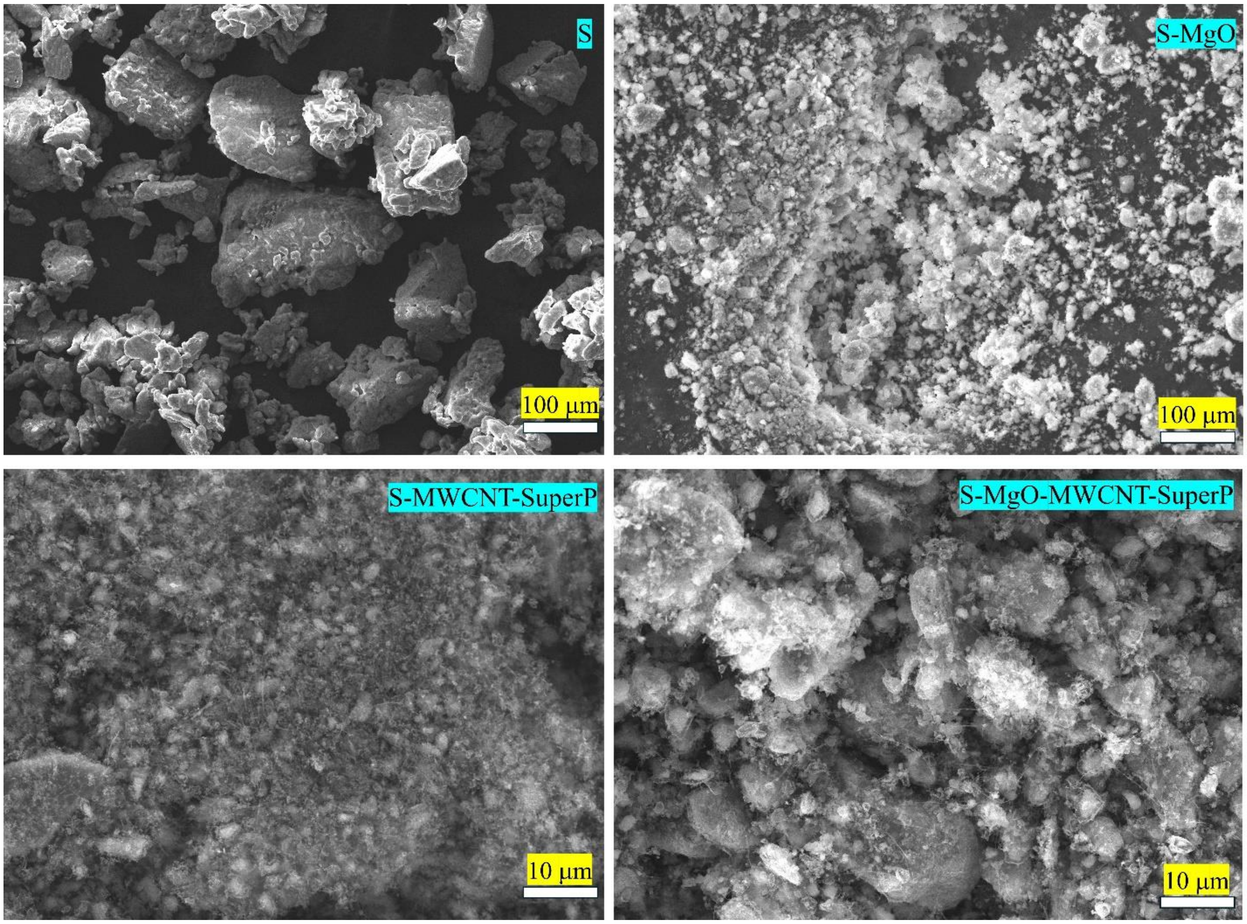

Figure S3 *SEM Images of Sulfur pristine to various composites*

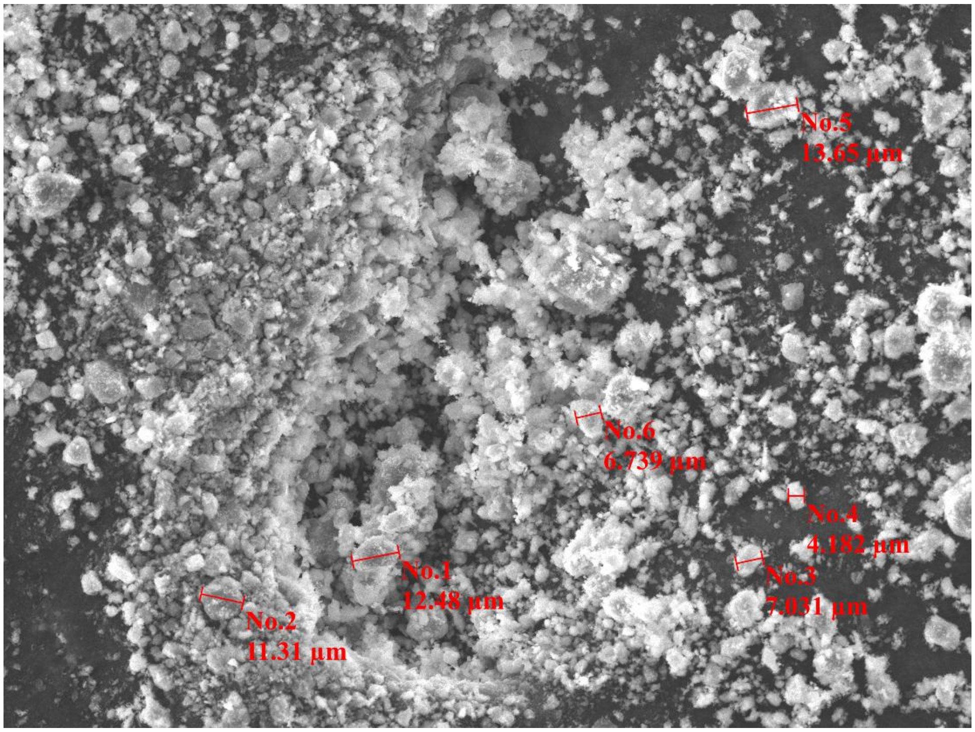

Figure S4 *SEM image of S-MgO*

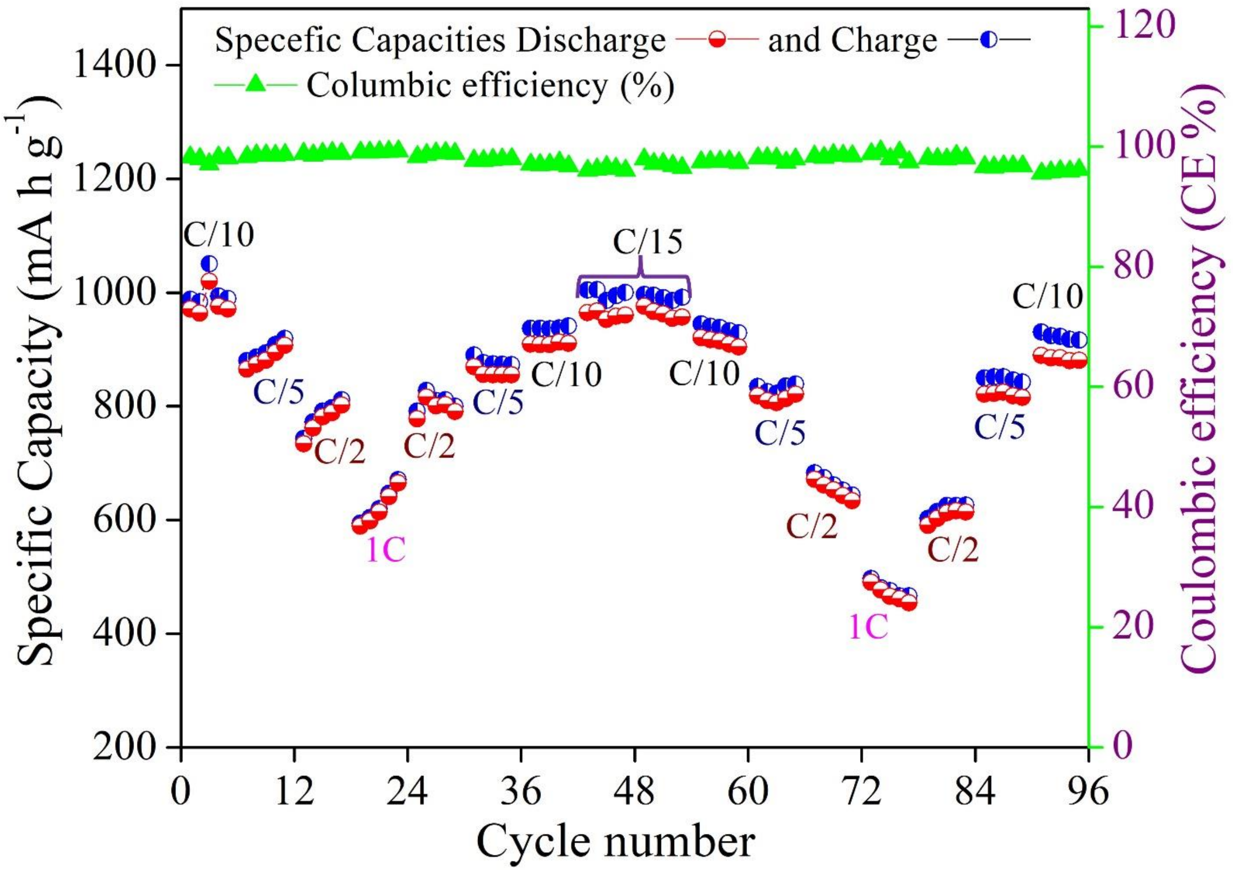

Figure S5 *Rate performance of Li-S coin cell (repetition with continue cycling)*

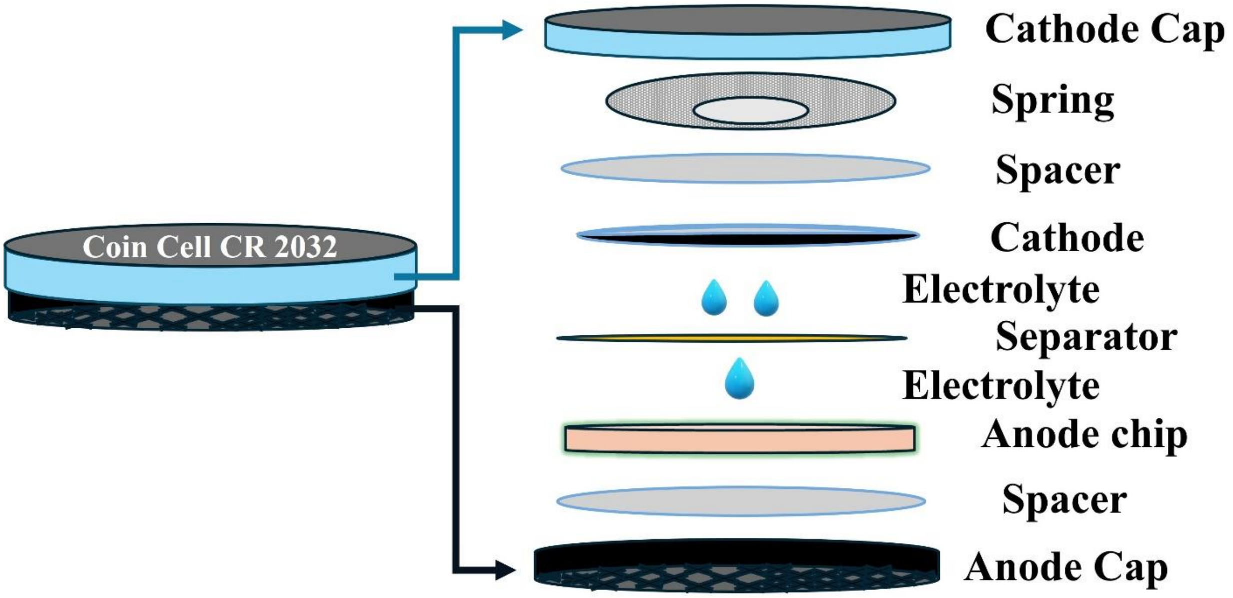

Figure S6 *Assembly of coin cell 2032 to fabricate the Li-S batteries utilized in the experiments*

Reference:

1. Domingo, C.; Montero, S. Raman intensities of sulphur α-S_8. *J. Chem. Phys.* **1981**, *74*, 862–872. https://doi.org/10.1063/1.441137.
2. Ward, A.T. Crystal-field splitting of fundamentals in the Raman spectrum of rhombic sulfur. J. Phys. Chem. 1968, 72, 744–746. https://doi.org/10.1021/j100848a063.
3. Nakamoto, K. Infrared and Raman Spectra of Inorganic and Coordination Compounds, Part B Applications in Coordination, Organometallic, and Bioinorganic Chemistry, 6th ed.; Wiley: Hoboken, NJ, USA, 2009. https://doi.org/10.1002/9780470405888.
4. Steudel, R. *Elemental Sulfur and Sulfur-Rich Compounds II*; Springer: Berlin/Heidelberg, Germany, 2003; Volume 248.
5. Eckert, B.; Steudel, R. Molecular spectra of sulfur molecules and solid sulfur allotropes. In *Elemental Sulfur und Sulfur-Rich Compounds II. Topics in Current Chemistry*; Springer: Berlin/Heidelberg, Germany, 2003; Volume 231, p. 3198. https://doi.org/10.1007/b13181.
